# Sleep in COVID-19 recovery period and its impacts on Quality Of Life

**DOI:** 10.1192/j.eurpsy.2022.1236

**Published:** 2022-09-01

**Authors:** S. El Sayed, S. Gomaa, A. Eissa, D. Shohry, A. Kabil

**Affiliations:** 1 Faculty of Medicine-Mansoura University, Department Of Psychiatry, faculty Of Medicine, Mansoura University, Mansoura, Egypt; 2 Faculty of Medicine-Port Said University, Department Of Neuropsychiatry, port Said University, Portsaid, Egypt; 3 Faculty of Medicine, Mansoura University, Community Medicine, Mansoura, Egypt; 4 Faculty of Medicine, Al Azhar University, Pulmnology, Cairo, Egypt

**Keywords:** sleep, sleep, covid-19, quality, life, quality, Covid-19

## Abstract

**Introduction:**

According to the World Health Organization (WHO), the COVID-19 infection became a worldwide devastating health issue starting in December 2019 in China and then gradually was a global pandemic. PTSD after recovery from COVID-19 has been correlated to sleep problems, high anxiety level and depressive manifestations. These sleep problems have their drastic effect on the recovered patients’ quality of life including physical, psychological and social domains.

**Objectives:**

1-To investigate the sleep in the post Coronavirus -19 period 2-If has an impact on the different items of patients’ quality of life.

**Methods:**

1-Socio-demographic characteristics of 500 recovered COVID-19 patients 2-Insomnia Severity index a brief scale evaluating the patient’s insomnia. The ISI evaluates the subjective complaints and results of insomnia as well as the level of dysfunctions from these sleep disturbances 3-Pittsburgh sleep quality index (PSQI):The Pittsburgh Sleep Quality Index (PSQI) is a scale that study the subjective sleep quality and different domains of sleep over a period of 1-month 4-Quality Of Life (QOL) by the SF36 Health Survey is a 36-item -report survey that evaluate eight domains of physical and mental wellbeing ranging from 0 to 100.

**Results:**

The mean score of insomnia severity index was 13.01±4.9.Regarding Pittsburgh sleep quality index ,Sum of seven component scores was 15.37±4.43.Also QOL SF36 showed higher scores of the 8 domains including physical and mental

**Conclusions:**

High score of insomnia and sleep disturbances during the recovery period of COVID-19 infection which affecting the Quality Of Life

**Disclosure:**

No significant relationships.

